# Mixed aerobic-anaerobic incubation conditions induce proteolytic activity from in vitro salivary biofilms

**DOI:** 10.1080/20002297.2019.1643206

**Published:** 2019-07-25

**Authors:** Leanne M Cleaver, Rebecca Moazzez, Guy H Carpenter

**Affiliations:** a Centre for Host Microbiome Interactions, King’s College London Faculty of Dentistry, Oral and Craniofacial Sciences, London, UK; b Centre for Oral, Clinical and Translational Science, King’s College London Faculty of Dentistry, Oral and Craniofacial Sciences, London, UK

**Keywords:** Oral biofilm, proteolysis, salivary proteins, *16S rRNA* gene sequencing, nuclear magnetic resonance, confocal microscopy

## Abstract

Oral biofilms have not been studied using both metabolome and protein profiling concurrently. Bacteria produce proteases that lead to degradation of functional salivary proteins. The novel protocol described here allows for complete characterisation of *in vitro* oral biofilms, including proteolytic, metabolic, and microbiome analysis.

Biofilms were grown on hydroxyapatite discs from whole mouth saliva, using sterilised saliva as a growth-medium, in different growth environments. Salivary protein degradation was assessed from spent saliva growth-medium using SDS-polyacrylamide gel electrophoresis (SDS-PAGE), and metabolic activity by nuclear magnetic resonance (NMR). Discs were assessed for depth and coverage of biofilms by confocal laser scanning microscopy (CLSM), and biofilms were collected at the end of the experiment for *16S rRNA* gene sequence analysis.

There was a significant difference in biofilm viability, salivary protein degradation, and metabolites identified between biofilms grown aerobically and biofilms exposed to an anaerobic environment. Bacterial *16S rRNA* gene sequencing showed the predominant genus in the 7-day aerobic biofilms was *Streptococcus*, in aerobic-anaerobic and anaerobic 7-day biofilms *Porphyromonas*, and in aerobic-anaerobic and anaerobic 13-day biofilms *Fusobacterium*.

This data suggests new growth requirements and capabilities for analysing salivary biofilms *in vitro*, which can be used to benefit future research into oral bacterial biofilms.

The functionality of saliva depends on proteins. It is well known that bacteria produce proteases that can lead to reduced salivary protein function. Saliva contains over a thousand different proteins, but the most abundant includes α-amylase, cystatin, histatin, statherin, proline-rich proteins and mucin. One example of their importance is the protection of teeth from dietary acidity. Almost immediately after brushing, the proteins present in saliva form a conditioning film upon the tooth surface [1], known as the acquired enamel pellicle. The acquired enamel pellicle has been shown to provide a level of protection against dental erosion by inhibiting dietary acids from coming into contact with and demineralising the enamel. Moazzez and colleagues [2,3] showed that enamel pellicle has an influence on the effects of acid erosion on the tooth surface by comparing participants with dental erosion, healthy controls, and samples with no enamel pellicle. Healthy controls had increased microhardness and decreased roughness of the enamel compared to erosion patient samples and samples that lacked an enamel pellicle.

**Figure 3.**
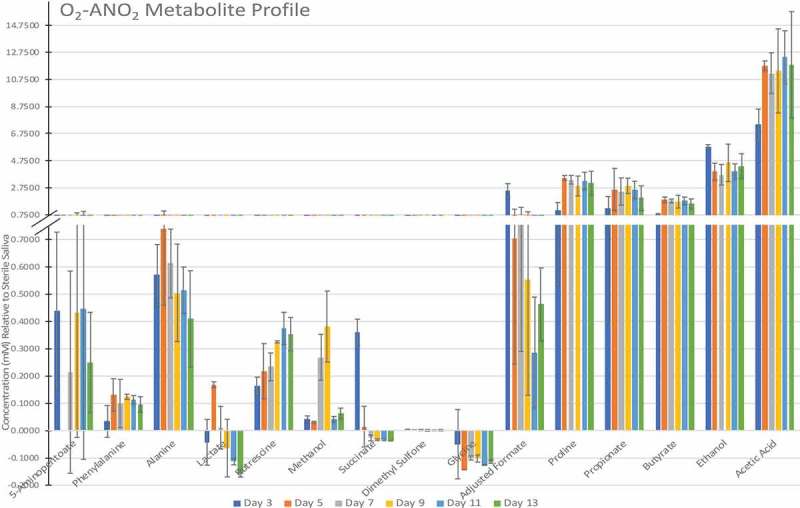
Metabolic profile of aerobic-anaerobically grown biofilms (mean ± SD, of three replicates, analysed once).

The oral cavity is rich in microorganisms, which includes archaea, bacteria (including the newly described candidate phyla radiation (CPR) [4], fungi, viruses and protozoa. The human oral cavity is made up of several different habitats which harbour distinct and characteristic biofilms. Bacteria that colonise the teeth first bind to host-specific molecules in the acquired enamel pellicle. It is thought that the early colonisers of the biofilm have a great impact on how the biofilm will further progress by what adherence molecules they lay down, and how this affects the oral health of the individual. Studies assessing the composition of the early oral biofilm have shown that the majority of early colonisers are aerobic or facultatively anaerobic species, such as *Streptococcus mitis, S. oralis, Actinomyces, Gemella, Granulicatella, Neisseria, Prevotella, Rothia*, and *Veillonella* [5,6]. It is pertinent to note that whilst *Veillonella* is an early colonising species, it cannot grow on its own and requires the metabolic capabilities of other bacteria [7], further suggesting that bacteria within a biofilm are symbiotic. *Fusobacterium nucleatum*, referred to as a so-called ‘bridge’ organism [8], is classed as a middle coloniser because it can coaggregate with early and late colonisers and is spatially found within the middle section in oral biofilm models [9,10]. Numerous coaggregation studies, reviewed by Kolenbrander and London [8], show that the final organisms to adhere to the maturing biofilm are predominantly facultative anaerobes/anaerobes such as *Aggregatibacter actinomycetemcomitans, Prevotella intermedia, Eubacterium* sp., *Treponema* sp., *Porphyromonas gingivalis* and *Selenemonas flueggei*. This, however, is not an exhaustive list, and is obviously limited heavily by the methods used in coaggregation studies, as it is known that there are species of oral bacteria that cannot be cultured. In contrast to these studies, there is new and conflicting evidence that suggests *Corynebacterium* is the most important bridging genus, and that plaque biofilms become more aerobic at the periphery as opposed to anaerobic [11]. Based on these differing coaggregation models, three different incubation environments were assessed in this study; purely aerobic, purely anaerobic and a mixed aerobic-anaerobic incubation.

Bacteria found in the oral cavity can produce proteases that contribute to oral disease, which offers an advantage to these bacteria, allowing them to proliferate and colonise surfaces of the mouth. The degradation of salivary mucins, largely by glycosidases, has been shown [3]. The most commonly recognised proteases are the gingipains produced by *P. gingivalis* in gingivitis and periodontitis; cysteine proteases Rgp and Kgp. Potempa et al. [12] showed that up to 85% of proteolytic activity within *P. gingivalis* is due to gingipains and these are a vital virulence factor for this organism. Immunoglobulin A1 (IgA1) protease has been identified in streptococci [13], serine proteases have been isolated from *Enterococcus faecalis* from root canal infections [14],, and a chemotrypsin-like protease is present in *Treponema denticola* [15] which allows the spirochete to invade the basement membrane of oral epithelial cells to cause inflammation and tissue destruction. Proteases are therefore produced by oral bacteria as virulence factors to cause tissue damage but they also produce proteases to facilitate growth and proliferation. The peptides generated in saliva by proteases allow their characterisation by comparison to known proteases [16]. Proteins in saliva perform many functions, therefore if bacteria are degrading those proteins, then it is important to study these proteases. A robust and reproducible methodology for identifying protease activity by bacteria in *in vitro* oral biofilms, when combined with other parameters, is required.

There are many methods, substrates, environments, inocula and growth media that can be utilised to grow oral biofilms (Darrene and Cecile) [17]. Culturing oral biofilms *in vitro* can be difficult to achieve, and can often result in an over-simplification of the composition or structure of the biofilm, which is why whole mouth saliva will be used here as an inoculating fluid. To study the conditions that may promote protease production targeted at only the salivary proteins, saliva was used here as the inoculum and the growth medium, so that a broad salivary protein profile could be studied. A previous study using saliva as an inoculum and growth-medium using a microfluidics system successfully presented an *in vitro* oral biofilm model which has a species composition comparable to supragingival plaque [18]. Small amounts of saliva were used, which is ideal for sample collection, but is not ideal for downstream applications on the spent growth-medium.

The current study aimed to produce a methodology to simultaneously assess longitudinal growth, metabolic profile, proteolytic activity, and end-point microbial diversity of biofilms *in vitro* using whole mouth saliva inoculated onto hydroxyapatite discs, as a substitute for tooth surfaces. The use of whole mouth saliva as a growth medium was used to model and assess the degradation of salivary proteins by oral biofilms as a marker of proteolysis of the acquired enamel pellicle under different incubation conditions.

## Materials and methods

### Saliva sample collection

\This study was approved by the King’s College London Research and Ethics Committee, reference HR-17/18–6116. Eighteen informed participants who had given consent chewed on unflavoured paraffin gum and expectorated until 10 ml saliva (approximately 10–15 minutes). This was performed in the afternoon, at least 1 hour after eating and drinking, with the exception of water. Participants were self-reported as systemically and orally healthy (healthy volunteers), had not taken antibiotics in the last 3 months, and were a mix of both male and female. Saliva was stored at −80°C until required with a 1 ml aliquot saved for *16S rRNA* gene sequencing.

### Saliva processing

Saliva samples were centrifuged at 1,500 RPM for 15 minutes. The saliva supernatant was removed and pooled from all samples (approximately 180 ml), boiled for 20 minutes to sterilise the saliva and left to cool to room temperature; hereby referred to as sterile saliva.

The loose pellet from each sample was pooled and 35 ml of sterile saliva was added and vortexed vigorously to resuspend the pelleted material; referred subsequently as the pooled saliva inoculum. A 1ml aliquot of pooled saliva inoculum was also saved for *16S rRNA* gene sequencing.

### Inoculation and incubation of hydroxyapatite discs

There were three incubation environments tested, with each incubation environment having a duration of 13 days. Biofilms were assessed for depth and coverage, bacterial composition of the biofilms, protein analysis and metabolic analysis.

Hydroxyapatite (HA) discs (Himed Medical, USA) were inoculated with 1 ml of pooled saliva inoculum, horizontally in a sterile microtitre plate, in triplicate, for each experiment. One HA disc inoculated with 1 ml sterile saliva was also included for each experiment as a negative control.

Incubation environments were as follows: (1) aerobic; HA discs were incubated at 37°C in a 40 L aerobic incubator (GenLab Ltd, UK), (2) anaerobic; HA discs were incubated at 37°C in a MACS-MG-1000 anaerobic workstation (Don Whitley, UK), (3) aerobic to anaerobic switch; HA discs were incubated initially for 24 hours at 37°C in the aerobic incubator and then moved to the anaerobic chamber at 37°C for the remainder of the experiment.

### Biofilm depth and coverage analysis

After an initial 24-hour period of incubation, and subsequently at every 48-hour period, saliva was removed from the HA discs (this will hereby be referred to as spent saliva) and saved at −20°C for further testing. HA discs were washed twice with sterile PBS, then 50 μl of SYTO-9 and 50 μl propidium iodide (PI) resuspended in sterilse PBS (LIVE/DEAD™ *Bac*Light™ Bacterial Viability Kit, ThermoFisher Scientific, USA) were added to each disc, and discs were incubated for 15 minutes at room temperature prior to analysis with a DM-IRE2 confocal laser scanning microscope (Leica Microsystems Heidelberg GmbH, Germany). Discs were handled at all time points with sterile forceps, and viewed on sterile coverslips.

Five random images (from each quadrant and the middle of each disc) were captured at x 63 magnification using the Leica Microsystems Confocal Software (version 2.61 Build 1537). Images were analysed using ImageJ Software (version 1.52a) for binary image analysis to give percentage coverage.

The depth of biofilms was calculated using z-series stacking over five areas of each disc. Calibration of the z-height was performed using fluorophore beads of known size. Depth was recorded in micrometres (μm). Fresh sterile saliva was added to the discs and they were incubated further.

### Protein analysis

Spent saliva from each disc at each time point was centrifuged at 16,000 RPM for 5 minutes. The supernatant was saved and split into two aliquots; one sample for protein analysis and one for metabolic analysis. A 15 μl aliquot of sample was added to 5 μl of loading dye and 1 μl dithiothreitol, and 15 μl of this was loaded onto a 4–12% Bis-Tris ready-made polyacrylamide gel (Invitrogen, USA) and run for 32 minutes at 200 volts constant and 120 amps for one gel, 240 amps for two gels. Gels were stained with Coomassie brilliant blue R solution (Merck, Germany) for 20 minutes and de-stained in 10% acetic acid. Gel images were captured using the ChemiDoc (Bio-Rad, USA) and images were analysed using Image Lab (Bio-Rad) using relative quantity tools.

### Metabolic analysis

An aliquot of 500 μl of spent saliva supernatant from each time point for the 13-day experiment and the sterile saliva sample was each mixed with 125 μl of TSP buffer in a 5 mm nucleic magnetic resonance (NMR) tube (Bruker, Germany). The tubes were sealed and analysed at the NMR Centre, King’s College London, UK on a 600 MHz NMR spectrometer (Bruker) with the following parameters; a TCI (1H/19F)/13C/15N Prodigy Cryoprobe, a temperature of 298K, PROJECT pulse sequence, a spectral width of 20.8 ppm, 65,536 points in the free induction decay (FID), an acquisition time of 2.62 seconds, a delay of 4 seconds between 2 scans, a total of 128 scans, and the total delay of the T2 filter was 80 milliseconds. The spectra were processed on TopSpin (Bruker) with Fourier Transform with an exponential window of 0.3 Hz, then phase correction, baseline correction and calibration of the TSP peak to 0 ppm. The concentration of metabolites relative to the sterile saliva baseline were collected using Chenomix NMR Suite (Chenomix Ltd, Canada).

### *Bacterial DNA extraction, quantification and* 16S rRNA *sequencing*

Upon completion of the 7- and 13-day experiments, the three discs for each incubation environment were vortexed for at least 1 minute in sterile PBS and pooled prior to bacterial DNA extraction. The stored aliquots of participant and pooled saliva inoculum were allowed to thaw slowly at 4°C overnight. All samples were centrifuged at 16,000 RPM for 10 minutes. The supernatant was discarded and the pellet was treated with lysozyme (Merck) for 30 minutes at 37°C, to enhance extraction of Gram-positive bacteria DNA, followed by extraction using the GenElute Bacterial Genomic DNA Kit (Merck) according to the manufacturer guidelines.

Extracted DNA was quantified using the Qubit fluorometry platform (ThermoFisher Scientific) according to the manufacturer handbook, and analysed using the Qubit fluorometer (Version 4.0). Results were reported in nanograms per microliter (ng/μl).

Variable regions 1–2 of the *16S rRNA* gene were amplified by PCR using the 27F (YM modifications) and 338R primers [as previously described by 19], with unique barcodes and Illumina MiSeq (Illumina, USA) adapters. PCR amplicons were purified using the Applied Biosystems SequalPrep Normalization kit (Thermofisher), pooled to approximately 0.5 ng/μl, and sent to the Genome Centre, King’s College London, UK where they were sequenced using the Illumina MiSeq v3 2 × 250 bp flow cell for paired-end sequencing.

### Analysis of sequencing data

Analysis of sequences was performed using the ‘mothur’ software suite, version 1.40.5 [20] using the MiSeq standard operating procedure [21] at mothur.org (November 2018). Sequences that were greater than 350 bp with homopolymers greater than 8 bp were excluded. Once trimmed, sequences were aligned with the Human Oral Microbiome Database (version 13). A sequence dissimilarity distance of 0.015 was used to cluster sequences into operational taxonomic units (OTUs) to summarise the alpha diversity of the bacterial communities present in all samples. The size of the smallest library was used as a randomly selected subsample for all other samples (in this instance, 3,267 sequences) to perform alpha and beta diversity. The alpha diversity summarised number of sequences, percentage coverage, observed sequences, and the inverse Simpson’s diversity index [23]. Samples were grouped by type – saliva, pooled saliva, and biofilms split by 7- or 13-day incubation and environment. Results were visualised by principal coordinates analysis (PCoA) and heat map in R-Studio (version 1.1.477).

### Statistical analyses

Data were assessed for normality, and parametric or non-parametric tests were applied as appropriate. A two-way ANOVA with multiple comparisons and Bonferroni correction was used for analysis of the depth and coverage of biofilms and protein analysis. Metabolic profiles were assessed with Pearson’s correlation and k-means principle component analysis.

## Results

### Biofilm coverage and depth

#### Coverage

**Figure 1.**
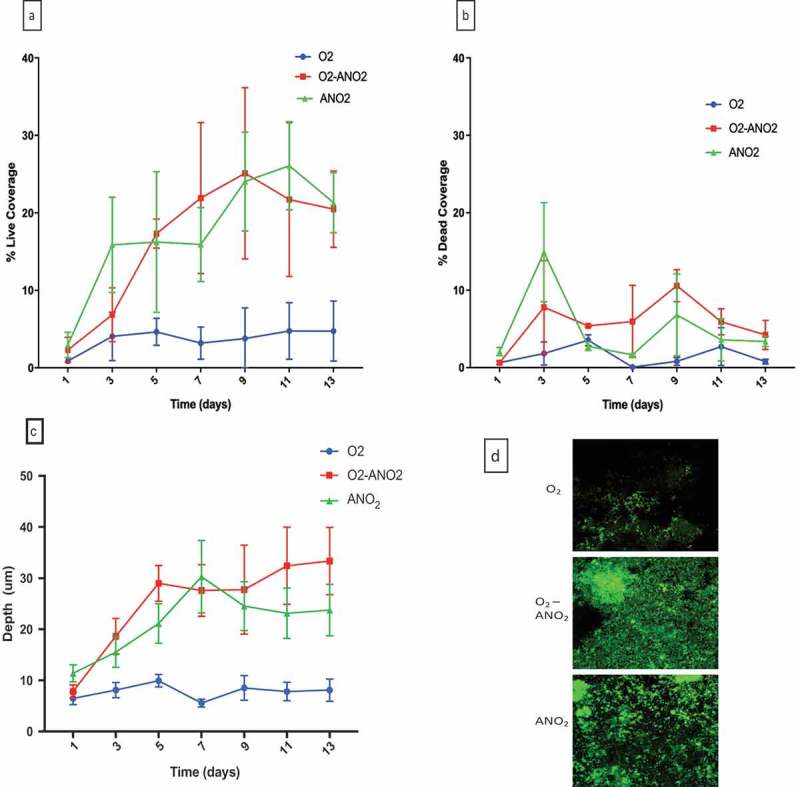
Depth and coverage of biofilms grown on HA discs over 13 days for each incubation environment (blue = aerobic, red = aerobic-anaerobic, green = anaerobic). (a); live percentage coverage, (b); dead percentage coverage, (c); depth of biofilms, (d); x 63 magnification CLSM images of live/dead viability staining of biofilms at day-13.

When assessed by live/dead viability, the negative control samples (no inoculum, sterile saliva) had a complete absence of bacterial growth. Figure 1(a) shows the coverage of live bacteria in biofilms adhered to HA discs. At days 5, 7, 9, 11 and 13 there was a significant difference (p = 0.049, 0.002, < 0.0001, 0.005, 0.01 respectively) between aerobic and aerobic-anaerobic biofilms, with aerobic-anaerobic biofilms having greater coverage of the discs. At days 7, 9, 11, and 13 there was a significant difference (p = 0.048, < 0.0001, < 0.0001, 0.007 respectively) between aerobic and anaerobic biofilms, with anaerobic biofilms having greater percentage coverage of the disc. There was no significant difference at any time point between aerobic-anaerobic and anaerobic biofilms.

Figure 1(b) shows the coverage of dead bacteria in the biofilms grown on HA discs. At days 3 and 9 there was a significant difference between aerobic and aerobic-anaerobic biofilms (p = 0.048 and 0.0005 respectively), and the same was true between aerobic and anaerobic biofilms (p = < 0.0001 and 0.046 respectively). There was a significant difference (p = 0.014) between aerobic-anaerobic and anaerobic biofilms at day 3. By the time the experiment was complete at 13 days there was no significant difference between all incubation environments for dead bacteria.

#### Depth

Figure 1(c) shows the depth of the biofilms in the three different environments. At days 3, 5, 7, 9, 11 and 13 there were significant differences (p = 0.015 at day 3, < 0.0001 for the remainder) between aerobic and aerobic-anaerobic biofilms, with aerobic-anaerobic biofilms having a greater average depth of biofilm. Anaerobic biofilms were significantly deeper than aerobic biofilms at days 5, 7, 9, 11 and 13 (p < 0.01 for days 5–13). There was no significant difference between anaerobic and aerobic-anaerobic biofilms until days 11 and 13, where aerobic-anaerobic biofilms were significantly deeper than anaerobic biofilms (p = 0.037 and 0.029).

### Metabolic analysis

**Figure 2.**
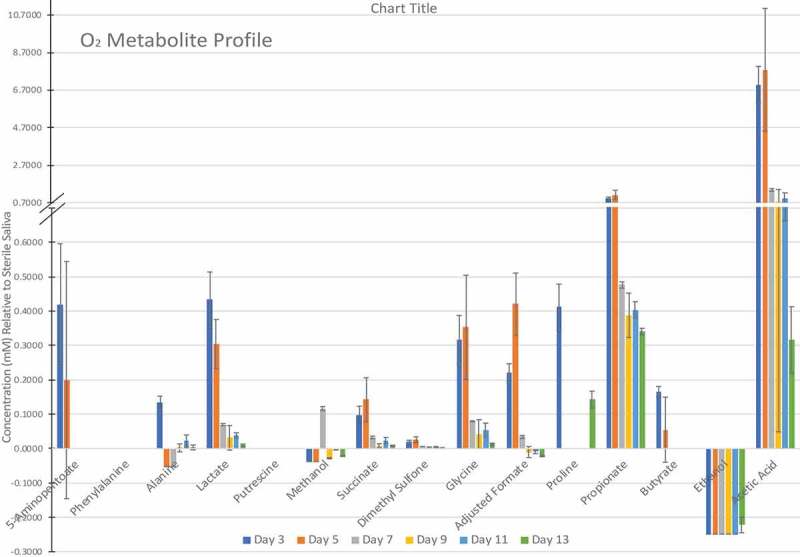
Metabolic profile of aerobically grown biofilms (mean ± SD, of three replicates, analysed once).

**Figure 4.**
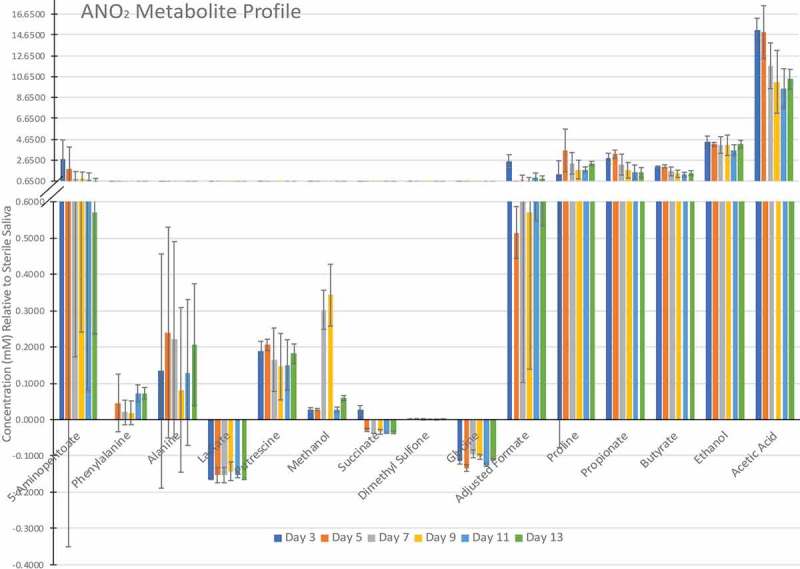
Metabolic profile of anaerobically grown biofilms (mean ± SD, of three replicates, analysed once).

Figure 2–4 show the metabolite profiles for the three incubation environments over the 13-day experiment relative to the sterile saliva medium, which showed reproducible differences. Acetate was produced in the highest quantities across all three incubation environments. There were significant differences in phenylalanine and putrescine produced when discs were incubated aerobically compared to anaerobic discs. There was a stable amount of 5-aminopentanoate produced by biofilms grown aerobic-anaerobically and anaerobically, however biofilms grown aerobically decrease to 0 mM after day 5. Of particular note was the high amounts of ethanol and proline present in the aerobic-anaerobic and anaerobic biofilms but only a small amount present in aerobic biofilms (at day 13, two-way ANOVA with multiple comparisons, p = < 0.0001 for both ethanol and proline between O_2_ and O_2_-ANO_2_/ANO_2_).

Glycine, lactate and succinate were present in lower quantities than the sterile saliva medium for aerobic-anaerobic and anaerobic biofilms suggesting utilisation by the biofilms. In contrast, only ethanol was significantly decreased compared to the sterile saliva medium in aerobically grown biofilms.

**Figure 5.**
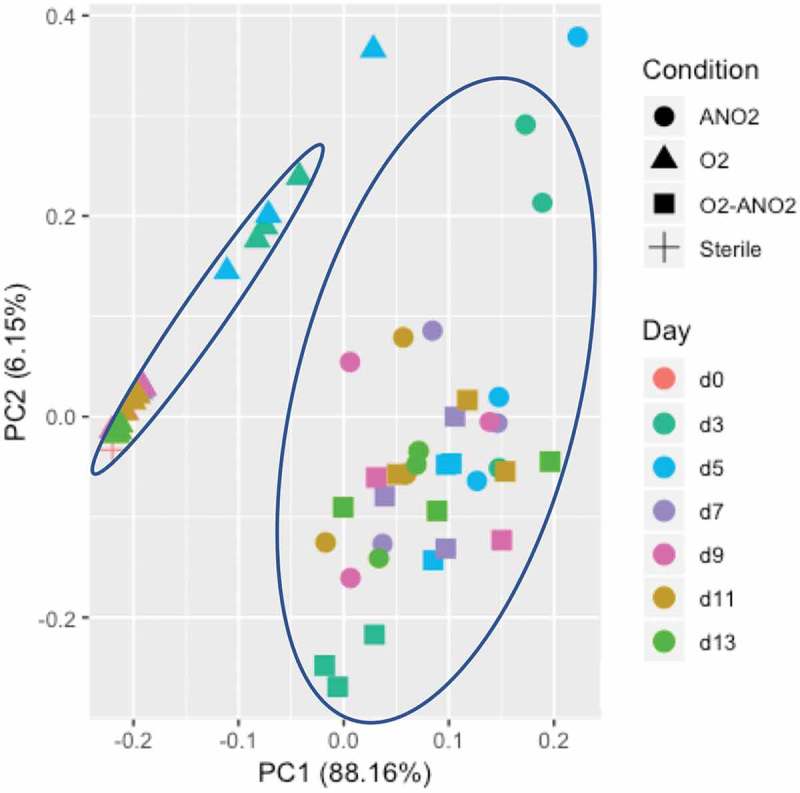
K-means principal component analysis for growth condition and time (days) of metabolites assessed by NMR. Two distinct clusters are circled in blue.

The principal component analysis of this data set shows that there were two distinct clusters within the data; one cluster for aerobic biofilms which clustered most closely to the sterile saliva sample, and one cluster for aerobic-anaerobic and anaerobic biofilms (Figure 5) which was distinct from the sterile saliva medium.

The increased production of phenylalanine, alanine and putrescine in the anaerobic and aerobic-anaerobically grown biofilms suggested these environments were inducing greater amounts of proteolysis. Since saliva contains many proteins, their relative abundance was characterised using SDS-PAGE separation with Coomassie Blue staining.

### Protein analysis

**Figure 6.**
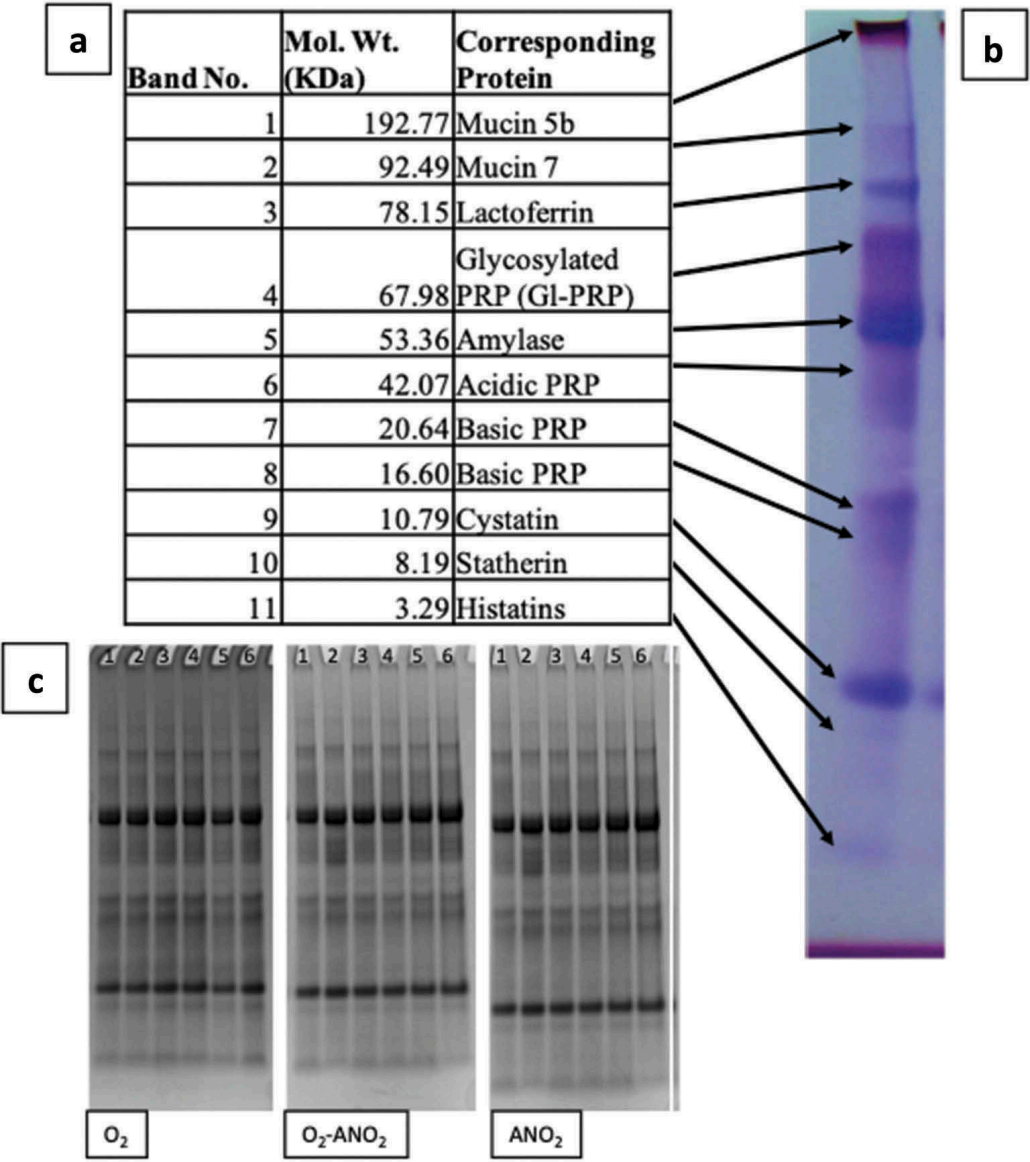
(a); 11 protein bands from the sterile saliva medium with their molecular weight and likely corresponding protein identification, (b); Coomassie-stained SDS-PAGE gel of the sterile saliva, (c); Coomassie-stained SDS-PAGE gels for aerobic, aerobic-anaerobic, and anaerobic control discs (lane 1 = day 3, 2 = day 5, 3 = day 7, 4 = day 9, 5 = day 11, 6 = day 13).

SDS-PAGE analysis of the sterile saliva medium revealed 11 distinct protein bands which were used as an inter-gel standard for relative quantification of each protein. The putative identity of the protein bands based on their molecular weight are presented in Figure 6(a).

The negative control discs for each incubation environment showed no protein degradation at any of the time points. Figure 6(c) shows the Coomassie stained gels for the control discs.

Table 1 shows the two-way ANOVA results for significant relative protein quantity of bands when samples are compared to the controls. Protein bands with non-significant data for all days have been excluded from Table 1. There was no significant difference between the controls and aerobic and anaerobic biofilms for bands mucin 5b (band 1), mucin 7 (band 2), lactoferrin (band 3) and histatin (band 11). A Wilcoxon signed rank test was performed for glycosylated PRP (band 4) as the data were non-parametric. All conditions were significantly different to the hypothetical mean of 1.00 (the relative quantity of the sterile saliva).

**Table 1.** Two-way ANOVA results for relative quantity of protein in samples compared to control samples for each condition on each day. Magnitude of change is shown as % change; a negative number shows a decrease in relative quantity of protein, and a positive number shows an increase in relative quantity when compared to the control.

O_2_	Significance											
	Day 3	% Change	Day 5	% Change	Day 7	% Change	Day 9	% Change	Day 11	% Change	Day 13	% Change
Band 5	Yes (0.0005)	-68	Yes (0.0097)	-49	Yes (0.0046)	-53	Yes (0.0113)	-53	Yes (0.0092)	-56	Yes (0.0131)	-47
Band 6	NS		NS		NS		NS		NS		Yes (0.0184)	-65
Band 7	Yes (0.0333)	-38	NS		NS		NS		NS		NS	
Band 10	Yes (0.0089)	119	NS		Yes (0.0470)	80	NS		NS		NS	
**O_2_-ANO_2_**												
Band 3	NS		Yes (0.0091)	-87	NS		Yes (0.0335)	-90	NS		NS	
Band 5	NS		Yes (0.0207)	-45	Yes (0.0030)	-56	Yes (0.0005)	-72	Yes (0.0162)	-52	NS	
Band 6	Yes (0.0150)	-63.00	Yes (0.0028)	-75	NS		Yes (0.0006)	-79	Yes (0.0064)	-77	Yes (0.0092)	-71
Band 7	Yes (0.0132)	-44.00	Yes (<0.0001)	-89	Yes (<0.0001)	-83	Yes (<0.0001)	-93	Yes (<0.0001)	-75	Yes (0.0034)	-55
Band 9	NS		NS		NS		Yes (0.0081)	-88	Yes (0.0310)	-81	Yes (0.0459)	-62
Band 10	NS		NS		NS		Yes (0.0309)	-94	NS		NS	
**ANO_2_**												
Band 5	NS		NS		NS		Yes (0.0319)	-46	NS		NS	
Band 6	NS		Yes (0.0248)	-59	NS		NS		NS		NS	
Band 7	Yes (<0.0001)	-75	Yes (<0.0001)	-83	Yes (0.0006)	-62	Yes (0.0021)	-57	Yes (0.0043)	-54	Yes (0.0224)	-44
Band 9	Yes (0.0120)	-79	Yes (0.0025)	-88	NS		NS		NS		NS	

**Figure 7.**
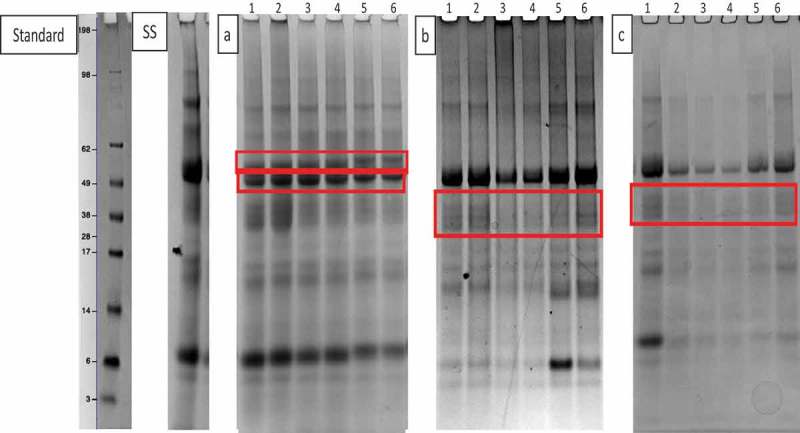
Coomassie-stained SDS-PAGE gels. Standard; SeeBlue Plus2 pre-stained standard, SS; Sterile saliva, (a); aerobically grown biofilms with the red box indicating the additional band above the amylase band, (b); aerobic-anaerobically grown biofilms with the red box indicating two additional bands within the acidic PRP region, (c); anaerobically grown biofilms with the red box indicating two additional bands within the acidic PRP region (lane 1 = day 3, 2 = day 5, 3 = day 7, 4 = day 9, 5 = day 11, 6 = day 13).

For the aerobic biofilms there was only one significantly different protein at all time points. Amylase (red box in Figure 7(a)) showed decreased intensity, but also a new band appeared with a slightly higher apparent molecular weight (60 kDa). For some other proteins, only transient changes were apparent; for basic PRP (band 6) there was a significant difference at day 13 only, for acidic PRP there was a significant difference at day 3 only, for statherin there was a significant difference at days 3 and 7, and there was no significant difference for basic PRP (band 8) or cystatin (band 9) at any time point.

The most differences were seen in the aerobic-anaerobic biofilms. Six bands were altered during incubation compared to three for aerobically grown films and four different proteins for anaerobically grown biofilms. Acidic PRP was significantly different at all time points. There was no significant difference at all time points for mucin 5b, mucin 7 and basic PRP. Lactoferrin (band 3) was significantly different at days 5 and 9, amylase at days 5, 7, 9 and 11, basic PRP was significant at all time points except day 7, cystatin was significant at days 9, 11 and 13, and statherin (band 10) was significant at day 9 only. There were two additional bands in the acidic PRP region of the gel on all three of the replicates (Figure 7(b), red box) that had an average molecular weight of 42 kDa and 35 kDa (disc 1; days 3, 5, 11 and 13, disc 2; days 3 and 9, disc 3; days 3, 7, 11 and 13). In contrast, incubation of the sterile saliva medium with the discs but without the saliva inoculum did not cause any alteration in protein abundance for any stained protein (see Figure 6(c)).

For the discs grown anaerobically, only one protein was altered significantly at all time points. Acidic PRP (band 7, 20 kDa) decreased in intensity at all time points compared to the sterile saliva. For amylase (band 5) there was a significant difference at day 9 only, for basic PRP (band 6) at day 5 only, and for cystatin at day 3 and day 5. There was no significant difference at any time point for basic PRP (band 8). There were two additional bands present on the protein gels within the acidic PRP region, with an average molecular weight of 38 kDa and 29 kDa, but this was only present on days 3, 5, 11 and 13 of the first replicate (Figure 7(c), red box).

### Sequencing analyses

All samples were sequenced, with the exception of the aerobic 13-day biofilm sample which failed to yield sufficient DNA for analysis. After quality filtering and removal of chimeras there were 206,290 sequences available for analysis. Table 2 shows the alpha diversity for all samples.

**Table 2.** Alpha diversity table showing richness and diversity of healthy volunteer (HV) and pooled saliva samples and biofilm samples from the three incubation environments.

Sample	Number of Sequences	% Coverage	Observed OTUs	Inverse Simpson Diversity Index
HV Pooled	6,510	87.70	1,113	36.11
HV001	3,267	90.36	498	23.20
HV002	4,934	90.49	688	41.08
HV003	4,972	93.75	466	13.84
HV004	8,159	94.36	693	18.00
HV005	8,513	94.82	701	12.49
HV006	5,176	94.32	446	6.74
HV007	9,967	93.91	926	19.35
HV008	8,723	93.66	800	10.78
HV009	11,827	94.77	925	18.71
HV010	8,125	92.66	891	21.48
HV011	9,320	95.08	703	7.34
HV012	9,039	93.56	895	36.74
HV013	9,215	94.48	848	46.96
HV014	6,755	93.83	637	18.32
HV015	4,409	95.44	318	10.66
HV016	3,461	90.87	498	38.86
HV017	9,562	93.54	946	18.44
HV018	10,327	94.90	803	16.61
Aerobic 7 day biofilm	5,052	94.20	464	25.23
Aerobic-anaerobic 7 day biofilm	13,556	96.02	819	13.27
Aerobic-anaerobic 13 day biofilm	14,819	97.77	512	4.74
Anaerobic 7 day biofilm	17,529	96.45	986	18.69
Anaerobic 13 day biofilm	13,073	97.21	524	5.90

**Figure 8.**
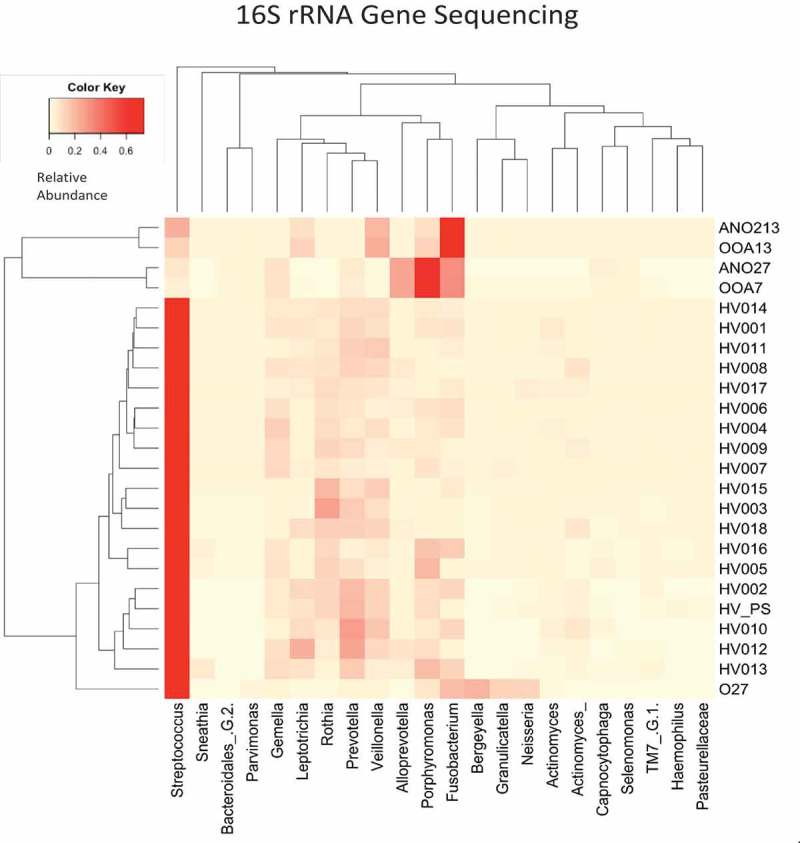
Heatmap chart showing the genus relative abundance for biofilm, saliva and pooled saliva samples plus a dendrogram to demonstrate the relatedness of samples. O27 = aerobic 7-day biofilm, OOA7 = aerobic-anaerobic 7-day biofilm, ANO27 = anaerobic 7-day biofilm, OOA13 = aerobic-anaerobic 13-day biofilm, ANO213 = anaerobic 13-day biofilm, HV_PS = healthy volunteer pooled saliva.

**Figure 9.**
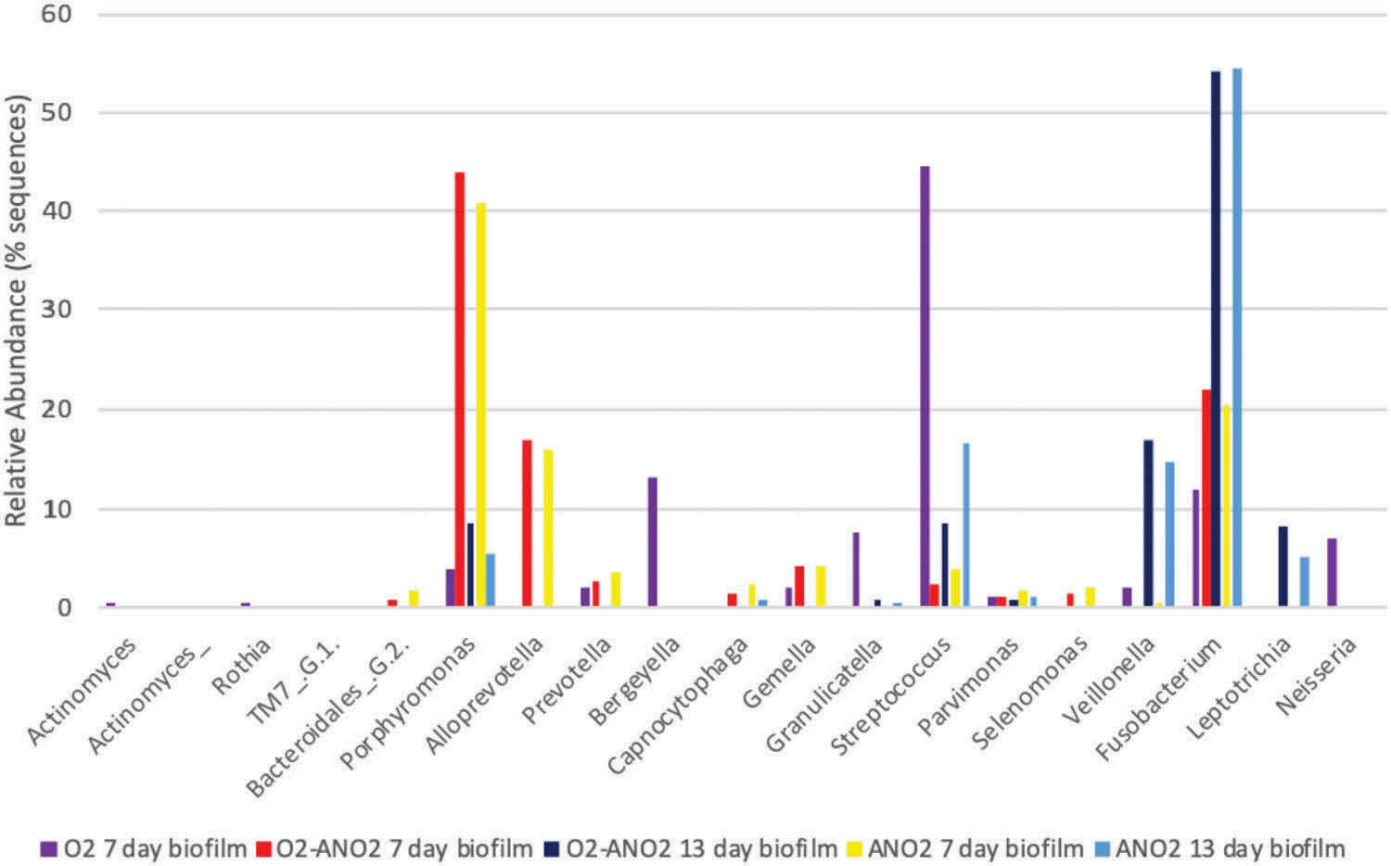
Diversity of 7- and 13-day biofilms based on relative abundance of genera within biofilms grown in different incubation environments.

The healthy volunteer saliva samples were individually included in the microbiome analysis to ascertain how closely related the individual saliva samples, the pooled saliva inoculum sample and the biofilm samples were. The healthy volunteer samples were also individually included to observe whether any of the individuals that had delivered the pooled saliva sample were skewing the bacterial community with a high abundance of one genus. The composition of the samples at genus level based on the highest relative abundance can be seen in the heatmap (Figure 8). There is a high abundance of *Streptococcus* in the healthy volunteer saliva samples, the pooled saliva inoculum sample, and the aerobic biofilm sample, and a lower abundance in the 13-day aerobic-anaerobic and anaerobic biofilm samples. There was a low abundance of *Streptococcus* in the aerobic-anaerobic and anaerobic 7-day biofilms. The predominating genus (and predominating species within that genus) in the 13-day aerobic-anaerobic and anaerobic biofilms was *Fusobacterium (nucleatum* subspecies *polymorphum)*, followed by *Veilonella (parvula), Leptotrichia (*not classified) and *Porphyromonas (catoniae)*. The predominating genus in the 7-day aerobic-anaerobic and anaerobic biofilms was *Porphyromonas (catoniae*), followed by *Alloprevotella (*unclassified) and *Fusobacterium (*aerobic-anaerobic; *nucleatum* subspecies *polymorphum*, anaerobic; *periodonticum)*. Table 3 demonstrates the most abundant species for each of the biofilm samples and the pooled saliva sample, based on number of total reads per sample. The dendrogram within Figure 8 shows the phylogenetic tree for all saliva and biofilm samples, demonstrating the clustering of samples based on microbial communities. The aerobic-anaerobic and anaerobic 7-day biofilms clustered closely together, along with the aerobic-anaerobic and anaerobic 13-day biofilm samples. All of the healthy volunteer saliva samples clustered together, along with the pooled healthy volunteer saliva sample. Figure 9 demonstrates the similar diversity of 7-day and 13-day biofilms based on incubation environment and relative abundance of genera.

**Table 3.** The five most abundant species of bacteria in the pooled saliva sample and the biofilm samples.

Sample	Genus/Species/Subspecies	No. of Reads	Relative Abundance in Sample (%)
Pooled Saliva	*Streptococcus* unclassified	2,369	36.4
	*Rothia mucilaginosa*	396	6.1
	*Prevotella melaninogenica*	352	5.4
	*Porphyromonas* unclassified	297	4.6
	*Streptococcus mitis*	290	4.5
Aerobic 7- day Biofilm	*Streptococcus* unclassified	1,224	24.2
	*Bergeyella* unclassified	593	11.7
	*Streptococcus* species oral taxon 071	421	8.3
	*Fusobacterium nucleatum* (subspecies *polymorphum*)	378	7.5
	*Granulicatella adiacens*	360	7.1
Aerobic-anaerobic 7- day Biofilm	*Porphyromonas* unclassified	2,882	21.3
	*Porphyromonas catoniae*	2,173	16.0
	*Fusobacterium nucleatum* (subspecies *polymorphum*)	1,632	12.0
	*Alloprevotella* species oral taxon 912	1,059	7.8
	*Fusobacterium periodonticum*	926	6.8
Aerobic-anaerobic 13- day Biofilm	*Fusobacterium nucleatum* (subspecies *polymorphum*)	6,888	46.5
	*Veillonella parvula*	2,480	16.7
	*Porphyromonas catoniae*	1,284	8.7
	*Leptotrichia* species oral taxon 212	886	6.0
	*Streptococcus* unclassified	615	4.2
Anaerobic 7- day Biofilm	*Porphyromonas catoniae*	4,484	25.6
	*Porphyromonas* unclassified	1,634	9.3
	*Fusobacterium nucleatum* (subspecies *polymorphum*)	1,608	9.2
	*Alloprevotella* unclassified	1,547	8.8
	*Fusobacterium periodonticum*	1,403	8.0
Anaerobic 13- day Biofilm	*Fusobacterium nucleatum* (subspecies *polymorphum*)	5,249	40.2
	*Veillonella parvula*	1,798	13.8
	*Streptococcus* species oral taxon 071	1,304	10.0
	*Fusobacterium* unclassified	773	5.9
	*Porphyromonas catoniae*	723	5.5

**Figure 10.**
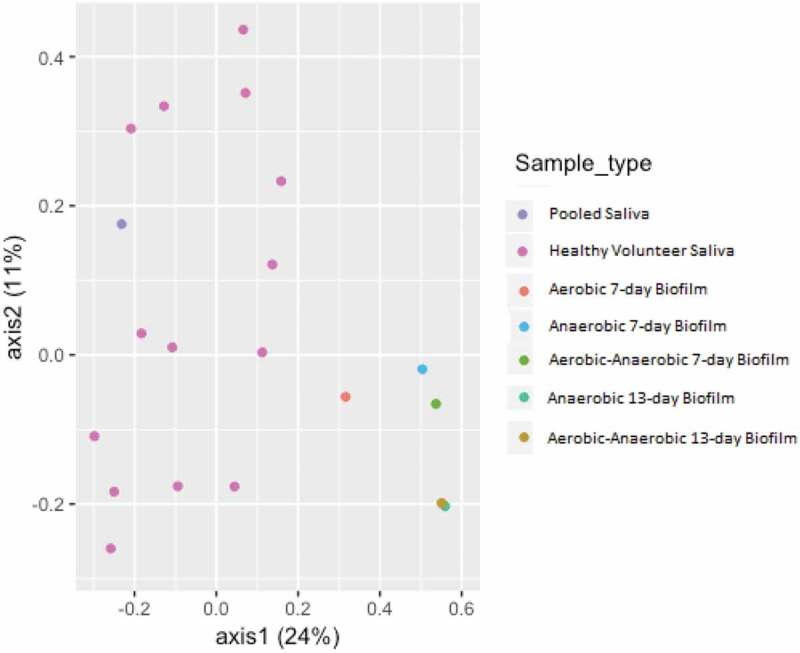
Principal coordinates analysis (PCoA) plot of pooled saliva, healthy volunteer saliva, and biofilm samples based on the Jaccard index of microbial communities.

The comparison of healthy volunteer saliva samples and the pooled saliva sample was assessed using principal coordinates analysis (PCoA, Figure 10). The plot shows that the saliva samples clustered closely together, with the aerobic 7-day biofilm sample clustering more closely to these samples than the biofilm samples. The 7-day aerobic-anaerobic and anaerobic biofilm samples clustered closely together, and the 13-day aerobic-anaerobic and anaerobic biofilm samples clustered closely together. Biofilms that are inoculated with the same saliva therefore show differences based on incubation environment and length of incubation time.

## Discussion

This study has successfully developed a method for assessing the proteolytic and metabolic activity of *in vitro* salivary biofilms, using saliva as an inoculum and growth medium. The use of whole mouth saliva as a conditioned medium is a beneficial approach to growing oral biofilms and accommodates the ability to test multiple parameters of biofilm growth. This method overcomes the limitations of other studies, where single- or multi-species biofilms have been grown using chosen bacteria, which may not reflect the actual conditions and symbiosis of the bacterial biofilm *in situ*. This is the first known study to report the use of NMR to analyse metabolites produced by oral biofilm bacteria grown in sterile whole mouth saliva.

Depth and coverage of hydroxyapatite discs shows that, while biofilms will grow in all incubation environments, anaerobic atmosphere caused the greatest growth. Kollenbrander et al.[7, 8, 9], have previously shown the early colonisers within the mouth are aerobic species and the middle and late colonisers are anaerobic species, which may account for the significant differences in coverage and depth in this study. The initial aerobic incubation step prior to transfer to an anaerobic environment significantly increased biofilm growth, but did not affect the bacterial diversity of the biofilms, as the biofilms exposed to purely an anaerobic environment were closely related. However, due to the *16S rRNA* gene sequencing only being performed on one end-point biofilm replicate for each condition, the statistical significance of this would require a greater number of samples. Additional replicates would necessitate a larger volume of saliva, as obtaining the required level of extracted DNA for sequencing involves pooling three replicates, which is a limitation to this study which already required 180 ml stimulated whole mouth saliva.

Another limitation to this experiment is that this method has not accommodated a quantifiable measure of biomass of the biofilms. The coverage and depth figures were acquired when looking at five random points on the HA disc, and may not be representative of biomass. Further experimentation will use previously published methods of wet-weighing HA discs before and after biofilm growth [24].

The degradation of proteins and the appearance of protein bands of lower molecular weight indicates the possibility of proteolytic activity by the bacteria in the biofilms. The greatest loss of salivary proteins was seen in the mixed aerobic-anaerobic incubation environment. It could be argued that the degradation is due to proteases already present in the saliva (mammalian origin), as one study has shown that over time three proteins with the molecular weights 2,937 Da, 3,370 Da and 4,132 Da appear which are the breakdown products of larger proteins [25]. However, since the inoculum was replaced and refreshed after 48 hours, unless these proteases attached to the hydroxyapatite they would not be present unless secreted by the biofilm. No changes in the protein bands were witnessed in the control discs (no biofilm growth), so auto-degradation of the sterile saliva is minimal. The additional protein band present in the aerobically grown discs at 60 kDa is likely to be a protein that is already present in the saliva, but obscured by the dense amylase band in the sterilised saliva, which becomes apparent as the quantity of this protein decreases. In all incubation environments there was no evidence of mucin degradation, of either mucin 5b or mucin 7. Previous studies have shown that mucin plays a role in the survival of biofilms, particularly of *S. mutans* [26], when there are no free amino acids available.

Using SDS-PAGE to detect loss of proteins in saliva samples is a quick and relatively inexpensive way of assessing the effects of incubation environment on salivary protein degradation by *in vitro* oral biofilms. Protein analysis will be studied in greater detail in future experiments by mass spectrometry and analysis of peptides and proteases using human and bacterial databases, and under different growth conditions.

In addition to the proteolytic assessment this study has also assessed the metabolic profile of sterile saliva before and after incubation with the biofilm. Other studies have used NMR directly from saliva for metabolic profiling of different oral conditions [1,27–29], but not from biofilms grown from a saliva inoculum. There are some limitations to this model. This was a one-dimensional analysis of the metabolites present, which gives less well-defined peaks, some of which are so broad they can mask the narrower peaks present. Two-dimensional NMR is available, where the spectra are further separated, which can provide greater detail and specificity for peaks, but requires more time to complete. This method may be utilised in future experiments.

An interesting result is the high concentration of ethanol in the aerobic-anaerobic and anaerobic biofilms and the low concentration of ethanol in aerobic biofilms, suggesting that biofilms that are exposed to anaerobic environments are ethanol producers and those not exposed are ethanol consumers. It is possible that this is due to bacterial or fungal fermentation of sugar in the absence of oxygen, which would explain the lack of ethanol in the aerobic biofilms. However, the sterilised saliva did not contain an excess of sugar, and participants did not eat or drink one hour prior to sample collection. It has been reported that bacteria produce alcohol dehydrogenase, reducing ethanol to acetaldehyde, which is subsequently oxidised to acetate [30], therefore the high levels of ethanol in these samples could be in part due to a lack of alcohol dehydrogenase. There was no available free-sugar in the sterile saliva for bacteria to utilise, however we have shown here that there was a breakdown of glycosylated proteins and proline rich proteins (higher levels of alanine and proline in the aerobic-anaerobic and anaerobic biofilms), which could provide sugar moieties for fermentation.

Short chain fatty acids were also produced only in the aerobic-anaerobic and anaerobic biofilms, which could be due to the fermentation of sugar by anaerobic species that are not present (*Porphyromonas*), or are less abundant (*Fusobacterium*), in the aerobic biofilms.

The production of lactate and glycine by the biofilms tested here was of interest, due to the higher levels in aerobic biofilms compared to the biofilms that were exposed to an anaerobic environment. It is well documented that with increased growth of the facultative anaerobe *S. mutans*, high levels of lactic acid is produced which contributes to caries development [31]. It has also been shown that *Veillonella*, an anaerobic species, utilises lactate under anaerobic conditions [32], and is thought to be a bridging molecule allowing the polymicrobial interaction of early and late (mostly anaerobic) colonisers in oral biofilms [7]. These results suggest that lactate and glycine are substrates required for increased biofilm growth. The levels of lactate identified by NMR were lowest at day-13 in the aerobic-anaerobic and anaerobic biofilms, where there was a moderately high relative abundance of *Veillonella*, which supports this theory.

The high levels of alanine and proline in the aerobic-anaerobic and anaerobic biofilms, coupled with the SDS-PAGE results showing increased degradation of proline rich proteins, along with the high abundance of anaerobes in these biofilms suggests that proteolytic activity could be due to facultative or obligately anaerobic species as opposed to aerobic species.

Based on the significantly greater biofilm growth and salivary protein degradation in the aerobic-anaerobic biofilms, and that the most relatively abundant species in these biofilms mimic the published Kolenbrander oral biofilm model, we suggest that when attempting to identify novel proteases that this aerobic-anaerobic *in vitro* oral biofilm method using saliva as an inoculum and growth medium is utilised.

The evident breakdown of salivary proteins will be investigated further in future experiments. Identification of the low molecular weight proteins present in spent saliva from biofilms using the novel and previously published endoProteoFASP method of protein detection [16] is planned. The crude nature of protein analysis here will be complemented by mass spectrometry to identify proteases present in the samples. This is alongside experiments to assess the effects of pulsing with varying compounds on proteolytic activity in salivary biofilms, particularly lactate, glycine and proline based on the NMR results presented here.

In conclusion, this novel method of characterising oral biofilms has successfully established a methodology to assess four different experimental parameters in one *in vitro* model. It also highlights the conditions in which proteases may be expressed, allowing for future analysis of salivary protein and acquired enamel pellicle degradation in different patient groups, such as in dental erosion. This study shows that there is a significant difference in degradation of salivary proteins and metabolic profile between biofilms that have been grown in an aerobic or anaerobic environment using the same saliva inoculum. It has also been observed that the microbial diversity of biofilms is driven by the incubation environment, regardless of identical inoculum. This data points to new growth requirements and capabilities for analysing salivary biofilms *in vitro*, which can be used to benefit future research into oral bacterial biofilms.
